# The Nature of Noradrenergic Volume Transmission From Locus Coeruleus to Brainstem Mesencephalic Trigeminal Sensory Neurons

**DOI:** 10.3389/fncel.2022.841239

**Published:** 2022-04-26

**Authors:** Hiroki Toyoda, Jonghwa Won, Wheedong Kim, Hayun Kim, Oscar Davy, Mitsuru Saito, Doyun Kim, Takuma Tanaka, Youngnam Kang, Seog Bae Oh

**Affiliations:** ^1^Department of Neuroscience and Oral Physiology, Graduate School of Dentistry, Osaka University, Suita, Japan; ^2^Department of Brain and Cognitive Sciences, College of Natural Sciences, Seoul National University, Seoul, South Korea; ^3^Department of Neurobiology and Physiology, School of Dentistry and Dental Research Institute, Seoul National University, Seoul, South Korea; ^4^Interdisciplinary Program for Brain Science, College of Natural Sciences, Seoul National University, Seoul, South Korea; ^5^School of Physiology, Pharmacology and Neuroscience, University of Bristol, Bristol, United Kingdom; ^6^Department of Oral Physiology, Graduate School of Medical and Dental Sciences, Kagoshima University, Kagoshima, Japan; ^7^Graduate School of Data Science, Shiga University, Hikone, Japan; ^8^Department of Behavioral Sciences, Graduate School of Human Sciences, Osaka University, Suita, Japan

**Keywords:** volume transmission, locus coeruleus, α_2A_-adrenergic receptor, mesencephalic trigeminal nucleus neurons, HCN channel

## Abstract

Noradrenergic neurons in the locus coeruleus (LC) release noradrenaline (NA) that acts *via* volume transmission to activate extrasynaptic G-protein coupled receptors (GPCRs) in target cells throughout the brain. As the closest projection, the dorsal LC laterally adjoins the mesencephalic trigeminal nucleus (MTN), in which proprioceptive primary sensory neurons innervating muscle spindles of jaw-closing muscles are exceptionally located. MTN neurons express α_2_-adrenergic receptors (α_2_-ARs) and display hyperpolarization-activated cyclic nucleotide-gated (HCN) currents (Ihs), which is downregulated by α_2_-AR activation. To quantify the activity-dependent outcome of volume transmission of NA from LC to MTN, we investigated how direct LC activation inhibits Ih in MTN neurons by performing dual whole-cell recordings from LC and MTN neurons. Repetition of 20 Hz spike-train evoked with 1-s current-pulse in LC neurons every 30 s resulted in a gradual decrease in Ih evoked every 30 s, revealing a Hill-type relationship between the number of spike-trains in LC neurons and the degree of Ih inhibition in MTN neurons. On the other hand, when microstimulation was applied in LC every 30 s, an LC neuron repeatedly displayed a transient higher-frequency firing followed by a tonic firing at 5–10 Hz for 30 s. This subsequently caused a similar Hill-type inhibition of Ih in the simultaneously recorded MTN neuron, but with a smaller Hill coefficient, suggesting a lower signal transduction efficacy. In contrast, 20 Hz activity induced by a 1-s pulse applied every 5–10 s caused only a transient facilitation of Ih inhibition followed by a forced termination of Ih inhibition. Thus, the three modes of LC activities modulated the volume transmission to activate α_2_-adrenergic GPCR to differentially inhibit Ih in MTN neurons.

## Introduction

It is well known that noradrenaline (NA) released from noradrenergic neurons in the locus coeruleus (LC) acts *via* volume transmission, which is the diffusion of neurotransmitter released at points that may be remote from the target cells with the resulting activation of non/extra-synaptic receptors with a much longer time course than those in synaptic transmission (Bach-y-Rita, 1993; Agnati et al., 1995; Zoli and Agnati, 1996; Taber and Hurley, 2014). Such a prolonged transmitter action, called tonic transmission, activates non-synaptic G-protein coupled receptors (GPCRs), in contrast to the phasic transmission that occurs rapidly at narrow synaptic cleft of single synapses (Agnati et al., 1995; Zoli et al., 1999; Farrant and Nusser, 2005; Sarter et al., 2009). LC neurons typically maintain a tonic spontaneous firing at 0.5–2 Hz, while LC neurons respond with phasic or burst firing at ∼20 Hz when the animal is exposed to salient stimuli, resulting in NA release in its target area such as the prefrontal cortex (PFC) (Florin-Lechner et al., 1996; Devoto et al., 2005). Such a differential activity of LC neurons may affect the outcome of volume transmission in target cells.

However, there has been no attempt to quantify or characterize the activity- dependent outcome resulting from GPCR activation and the subsequent signal transduction in target neurons following volume transmission from the source to target neurons. This is largely because the target pyramidal neurons in the PFC that have been most extensively studied in *in vitro* or *in vivo* preparations are located too remotely from the source LC neurons. Such a remote and diffuse projection makes it very difficult to find a functionally connected pair of LC and PFC neurons in *in vivo* preparations, and hence is also not suitable for simultaneously controlling and detecting the activity of LC neurons and the intracellular signal transduction in PFC pyramidal neurons, respectively. The closest projection site from LC may be the mesencephalic trigeminal nucleus (MTN), as MTN neurons receive dense noradrenergic fibers arising from the medially adjoining LC (Takahashi et al., 2010). In addition, non-synaptic α_2_-adrenergic receptor (AR) has been detected in MTN neurons (Aoki et al., 1994; Scheinin et al., 1994). In the brainstem MTN, there exceptionally exists proprioceptive primary sensory neurons that innervate muscle spindles of jaw-closing muscles, thereby receiving various synaptic inputs from the central nervous system (CNS) and onto diverse neurotransmitter receptors (Copray et al., 1990; Lazarov, 2002). It is known that MTN neurons display prominent hyperpolarization-activated, cyclic nucleotide-gated (HCN) channel currents (Ih) (Khakh and Henderson, 1998; Kang et al., 2004), and that HCN1/2 are robustly expressed in the soma of MTN neurons (Notomi and Shigemoto, 2004). Therefore, it may be possible that noradrenergic inputs from the LC participates in the modulation of Ih in MTN neurons by α_2_-AR activation (Copray et al., 1990; Scheinin et al., 1994; Rosin et al., 1996). Thus, the projection from LC to MTN maintained in *in vitro* slice preparations may be suitable to investigate the activity-dependent outcome of the volume transmission of NA.

In the present study, we addressed whether and how volume transmission of NA occurs from LC neurons to modify Ih in MTN neurons by performing dual whole-cell recordings from LC and MTN neurons. It was found that there was a non-linear relationship between the cumulative numbers of current pulses repetitively injected in LC neurons every 30 s to induce 20 Hz spike trains and the degree of inhibition of Ih in MTN neurons. This non-linear relationship obtained in response to the periodic phasic firings at 20 Hz was best described by the Hill equation with a Hill coefficient (nH) of approximately 1.5, which reflects the sensitivity to input spikes in LC neurons. However, the nH was variable depending on the mode of activity in LC neurons: smaller for the tonic firings at 5–10 Hz than for the periodic phasic firings at 20 Hz. In contrast, the periodic phasic firing evoked every < 10 s can forcibly terminate the Ih inhibition after transient enhancement of Ih inhibition. Thus, the volume transmission of NA from LC to MTN, accompanied by α_2_-adrenergic GPCR activation, is dynamically and differentially controlled by the three distinct patterns of firing activity of LC neurons.

## Materials and Methods

All experimental procedures were reviewed and approved by the Institutional Animal Care and Use Committee at Osaka University, Seoul National University, and by the institutional Animal Welfare and Ethical Review Body at University of Bristol. All experiments were performed in accordance with the relevant guidelines including the UK Animals in Scientific Procedures Act 1986.

### Procedure for Immunohistochemical Examination

In three C57BL6/J mice at postnatal day (PD) 21, noradrenergic projections from LC to MTN neurons were visualized by labeling with anti-tyrosine hydroxylase (TH) antibody and anti-advillin antibody, as advillin is known to be a marker for primary sensory neurons (Hasegawa et al., 2007). The mice were sacrificed by deep anesthesia with sodium pentobarbital (80 mg/kg, i.p.) and perfused transcardially with 5 ml of heparinized normal saline, followed by 10 ml of a freshly prepared 4% paraformaldehyde in 0.1 M phosphate buffer (PB; pH 7.4). The brainstem, including MTN and LC (–9 to –10 mm from bregma), was dissected out and immersed in 4% paraformaldehyde solution for overnight at 4°C.

For immunostaining in C57BL6/J mice, after cryoprotection with 30% (w/w) sucrose in phosphate buffered saline (PBS), the brainstem, including MTN and LC regions, was cut into 10 μm-thick coronal sections on a freezing microtome. Then, the sections were washed five times with PBS for 5 min each. For primary antibody treatment, the sections were incubated for 24 h at 4°C in PBS containing 0.5% (v/v) Triton X-100 and 4% (v/v) normal human serum with mouse anti-TH (1:500; sc-25269, Santa Cruz Biotechnology, Santa Cruz, CA, United States) or together with rabbit anti-advillin (1:200; ab72210, Abcam, Cambridge, MA, United States) or rabbit anti-dopamine beta hydroxylase (DBH) (1:500; 22806, ImmunoStar, Hudson, WI, United States). For secondary antibody treatment, the sections were treated with fluorescein isothiocyanate (FITC) donkey anti-mouse IgG (1:500; 715-095-151; Jackson ImmunoResearch) for TH and Cy3 donkey anti-rabbit IgG (1:500; 711-165-152; Jackson ImmunoResearch, West Grove, PA, United States) for advillin or DBH diluted in PBS for 2 h at room temperature (RT).

C57BL6/J mice (*n* = 5) were also used to examine the possible distribution of α_2A_-AR in MTN neurons. After cryoprotection with 30% (w/w) sucrose in PBS, the brains were cut into 12 μm-thick coronal sections on a freezing microtome. The sections were incubated for 24 h in PBS containing 0.5% (v/v) Triton X-100 and 4% (v/v) normal human serum with rabbit anti-advillin (1:500; ab72210, Abcam) and goat anti-α_2A_-AR (1:200; ab45871, Abcam). For secondary antibody treatment, the sections were treated with Cy3 donkey anti-rabbit IgG (1:500; 711-165-152; Jackson ImmunoResearch) and FITC donkey anti-goat IgG (1:500; 705-095-147; Jackson ImmunoResearch) diluted in PBS for 2 h at RT. The antigen retrieval was performed by placing the tissue in 95°C preheated citrate-based buffer (Vector Lab. Inc, Burlingame, CA, United States) for 20 min. For the histological identification of the recorded neurons after electrophysiological experiments (Karadottir and Attwell, 2006), the neurons were recorded with internal solution containing 0.1% Lucifer yellow (L1177; Thermo Fisher, Waltham, MA, United States) or 0.2% biocytin (B4261; MilliporeSigma, Billerica, MA, United States). After recording, the slices were immersed in 4% paraformaldehyde (P2031-1000; Biosesang, Seongnam, South Korea) in PBS overnight at RT. After washout of 0.1 M PBS, brain slices were frozen overnight in PBS containing 30% sucrose. The slices were washed with PBS and permeabilized in PBS containing 5% normal donkey serum and 0.5% Triton X-100 for 6 h in RT. For primary antibody treatment, the slices were treated with rabbit anti-TH (1:500, BML-SA497-0100; Enzo Life Sciences, Lausen, Switzerland) for overnight at 4°C. For secondary treatment and biocytin detection, the slices were treated with Cy3 donkey anti-rabbit IgG antibody (1:200, 711-165-152; Jackson ImmunoResearch) and Alexa Fluor 633-conjugated streptavidin (S21375; Thermo Fisher) diluted in PBS overnight at 4°C.

The slices were coverslipped with mounting media (H1000; Vector Laboratories, Burlingame, CA, United States), and immunostained neurons and neuropils were visualized with a confocal microscope (LSM700; Carl Zeiss, Oberkochen, Germany) at 10x, 63x, and 100x magnification.

### Slice Preparation

Slices were prepared from Sprague-Dawley rats (Figures 2–5 and Supplementary Figures 2, 4, 5) and C57BL/6 mice (Figure 6 and Supplementary Figure 3) of either sex at PD 14–21 by modifying previous methods (Chung et al., 2015). The animals were anesthetized with isoflurane, and the brains were quickly removed from the skull and immersed in ice-cold modified artificial cerebrospinal fluid (aCSF) containing the following (in mM): 210 sucrose, 1.8 KCl, 1.2 KH_2_PO_4_, 26 NaHCO_3_, 0.5 CaCl_2_, 2.5 MgCl_2_, and 50 D-glucose. With a microslicer (Vibratome 1,000 Plus; Leica), coronal sections of 250 μm thickness, including the MTN and LC, were cut and incubated at RT for 30 min in 50% modified aCSF and 50% normal aCSF (N-aCSF, pH 7.3) containing the following (in mM): 124 NaCl, 1.8 KCl, 1.2 KH_2_PO_4,_ 26 NaHCO_3_, 2.0 CaCl_2_, 1.0 MgCl_2_, and 10 D-glucose. The slices were then placed in N-aCSF at RT. N-aCSF was continuously gassed with a mixture of 95% O_2_ and 5% CO_2_.

**FIGURE 1:**
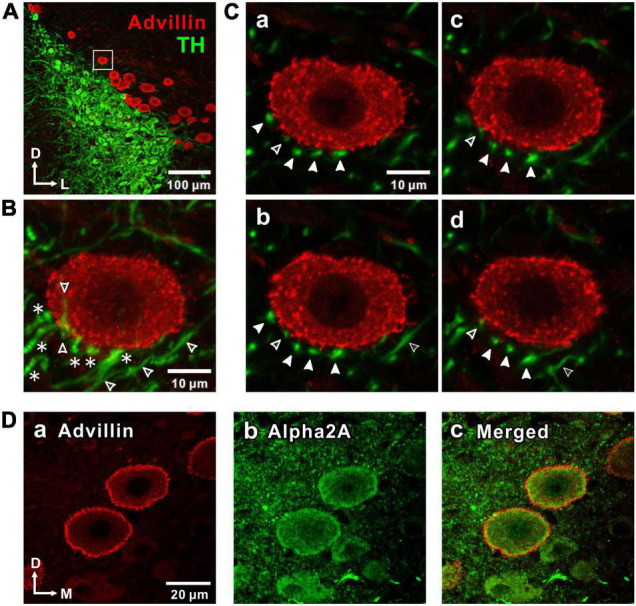
Noradrenergic projections from the locus coeruleus (LC) to the mesencephalic trigeminal nucleus (MTN) neurons and expression of α_2_-adrenergic receptor (α_2A_-Ars) in MTN neurons. **(A)** A confocal image (10x magnification) of a coronal brain section showing LC nucleus and MTN, in which LC neurons were immunostained with tyrosine hydroxylase (TH; green), while MTN neurons were immunostained with advillin (red). The region enclosed with a square was further visualized at 100x magnification as shown in a larger scale in **(B,C)**. **(B)** A Z-stack projection of 8 serial confocal images of the square region taken at 1 μm increments at 100x magnification. An advillin-positive MTN neuron is surrounded by TH-positive terminal-like swellings (filled arrowheads in **C**) arising from beaded axons (asterisks). Open arrowheads indicate TH-positive unbeaded axons. **(C)** Four serial single plane images (a: 2nd, b: 3rd, c: 4th, d: 5th) of those 8 serial ones. Filled arrowheads indicate the distribution of terminal swellings of LC axons. Open arrowheads indicate unbeaded axons (b,d). **(D)** Confocal images of MTN neurons showing the immunoreactivities for advillin (a) and α_2A_-ARs (b), together with a merged one (c).

**FIGURE 2:**
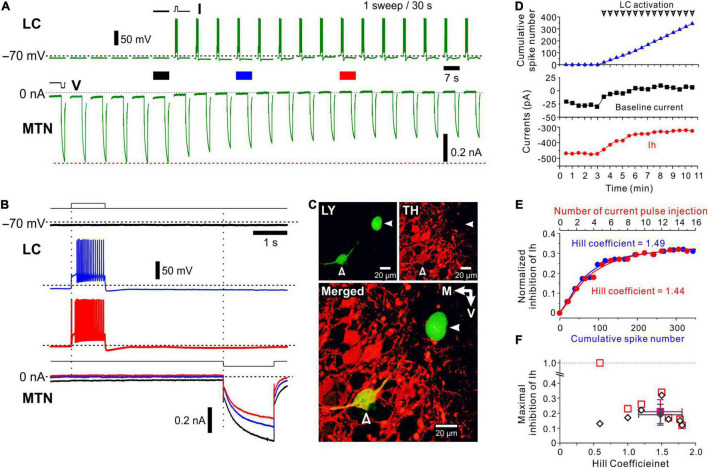
Effects of spiking of an LC neuron on hyperpolarization-activated, cyclic nucleotide-gated HCN channel current (Ih) evoked in a simultaneously recorded MTN neuron. **(A)** Following recording of six control Ih evoked in an MTN neuron, a depolarizing current pulse was applied every 30 s to induce a spike train approximately at 20 Hz in an LC neuron (top). Ih was repeatedly evoked in the MTN neuron every 30 s (bottom). In response to repeated depolarizing current pulses, amplitudes of Ih gradually decreased. **(B)** Superimposed enlarged Ih responses evoked in the MTN neuron seen during the respective time periods indicated with black, blue and red solid bars in **(A)** (top). Enlarged voltage responses evoked in the LC neuron during the respective time periods indicated in **(A)** (bottom). **(C)** Identification of simultaneously recorded LC and MTN neurons with Lucifer yellow (open and filled arrowheads, respectively; top left). Confocal photomicrographs showing immunoreactivity for TH (top right). A merged image showing that TH immunoreactivity was observed in the LC neuron (open arrowhead) but not in the MTN neuron (filled arrowhead; bottom). **(D)** Plotting of time and baseline current amplitude (black square), Ih amplitude (red circle), and cumulative spike number (blue triangle) before and during activation of LC neurons. Arrowheads indicate spike trains. **(E)** Plotting of cumulative spike number/current pulse injection and normalized inhibition of Ih. The blue curve was obtained by the Hill equation fitting to the data points for cumulative spike number while the red curve was obtained by the Hill equation fitting to those for current pulse injection. **(F)** Plotting of the Hill coefficient (nH) and maximal inhibition of Ih (*n* = 6). Filled diamond and square indicate mean values of nH and maximal inhibition in measured (open diamonds) and calculated (open squares) values, respectively.

**FIGURE 3:**
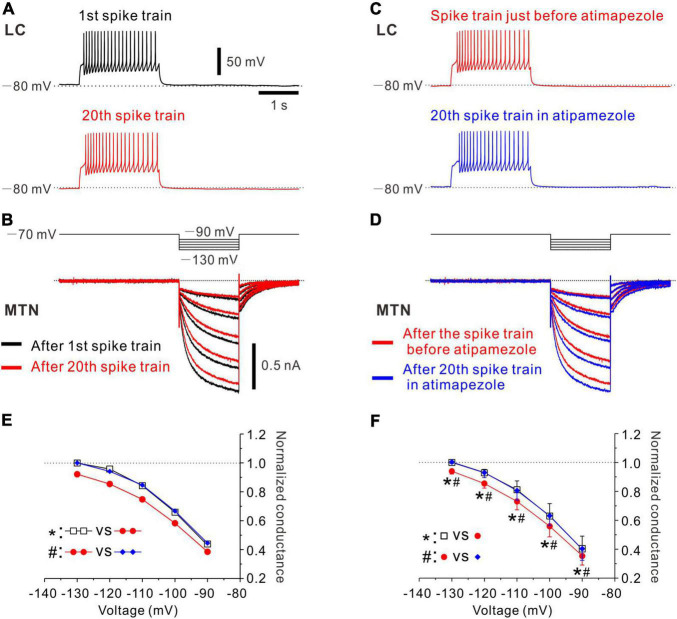
Effects of atipamezole on inhibition of Ih evoked in an MTN neuron by the spike activity of single LC neuron. **(A)** Black trace represents a train of spikes at 20 Hz which was induced in an LC neuron after recording of control Ih responses in an MTN neuron (1st spike train). Red trace represents 20th spike train. **(B)** Superimposed traces of Ih responses evoked by negative voltage pulses stepped from –70 to –90 up to –130 mV by 10 mV step in an MTN neuron obtained before 1st spike train (black trace), and those obtained after evoking 20th spike train (red trace) induced in a simultaneously recorded LC neuron. **(C)** Red trace represents the 20th spike train. Blue trace represents 20th spike train in the presence of atipamezole. **(D)** Superimposed traces of Ih responses evoked by negative voltage pulses obtained after evoking 20th spike train (red trace) and those obtained after evoking 20th spike train in the presence of atipamezole (blue trace). **(E)** Plotting of voltage and normalized conductance of Ih obtained before 1st spike train (black square), after 20th spike train (red circle), and after 20th spike train in the presence of atipamezole (blue diamond) in one paired recording. Two-way ANOVA: *p* < 0.001, followed by Bonferroni *post hoc* test, *p < 0.001, and #*p* < 0.001. **(F)** Plotting of voltage and normalized conductance of Ih in a total of four paired recordings. Two-way repeated measures ANOVA: *p* = 0.006, followed by Bonferroni *post hoc* test, *p < 0.002 (the largest *p*-value is 0.0015) and #*p* < 0.001 (the largest *p*-value is 0.0002).

**FIGURE 4:**
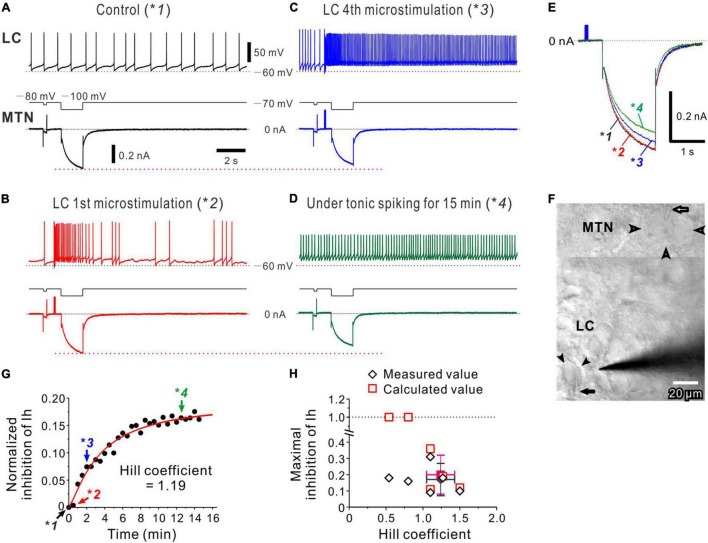
Noradrenaline (NA) volume transmission from multiple LC neurons to an MTN neuron. **(A)** Occurrence of spontaneous firing approximately at 0.8 Hz in an LC neuron, during which Ih was induced in an MTN neuron (*1). **(B)** The 1st microstimulation of LC inducing a transient increase in spike firing in the LC neuron without causing no marked inhibition of Ih evoked in the MTN neuron (*2). **(C)** The 4th microstimulation of LC inducing a persistent increase in spike firing and causing an apparent inhibition of Ih (*3). **(D)** Tonic spike firing approximately at 5–7 Hz in the LC neuron, causing a further inhibition of Ih (*4). **(E)** Superimposed traces of Ih responses indicated with *1, *2, *3, and *4 in **(A–D)**. **(F)** A composite photomicrograph; the upper and lower parts taken at a superficial focal plane to show an MTN neurons (open arrowheads) and at a deeper focal plane to show an LC neuron (filled arrowheads), respectively, from which dual whole-cell recordings were performed while microstimulation was applied to LC. Arrows indicate patch pipettes. **(G)** Plotting of time and normalized inhibition of Ih. Plots indicated with *1, *2, *3, and *4 were obtained from results shown in **(A–D)**. The red curve was obtained by the Hill equation fitting to the data points. **(H)** Plotting of maximal inhibition of Ih against nH and (*n* = 6). Filled diamond and square indicate mean values of nH and maximal inhibition in measured (open diamonds) and calculated (open squares) values, respectively.

**FIGURE 5:**
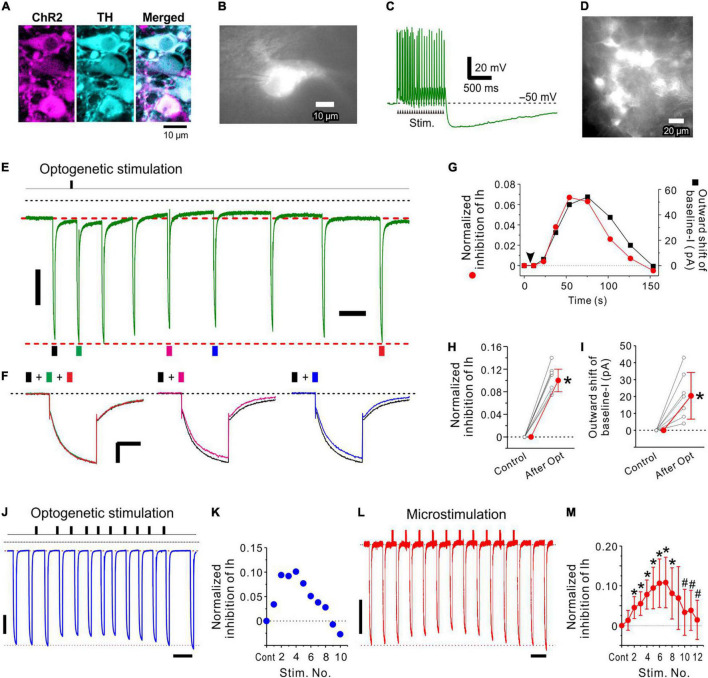
Optogenetic activation of LC neurons inhibits Ih evoked in an MTN neuron. **(A)** Photomicrographs showing mCherry and TH expression in LC neurons (left and middle, respectively) following transduction with CAV-PRS-ChR2-mCherry. A merged image of ChR2-mCherry and TH expression (right). Note that all mCherry-positive neurons were TH-positive neurons. **(B)** A ChR2-mCherry expressing LC neuron from which whole-cell recordings were obtained. **(C)** A train of > 20 spikes evoked by optogenetic stimulation (20 mW, 20 pulses of 4 ms duration at 20 Hz) of the LC neuron shown in **(B)**. **(D)** A photomicrograph obtained during optogenetic stimulation in a slice preparation including ChR2-expressing LC neurons. **(E,F)** In response to optogenetic stimulation of LC neurons (arrow), the amplitude of Ih evoked in an MTN neuron was slowly decreased (compare black trace with green, pink, and blue ones) and then slowly recovered to the original level (compare black trace with red trace). This change was accompanied by the outward shift of baseline currents. **(G)** Time courses of normalized inhibition of Ih (red circle) and outward shift of baseline currents (baseline-I; black square). The baseline current level is masked by the long-lasting tail current (tail-I) of the preceding Ih. Baseline current level just before the onset of each Ih was compensated by subtracting the amplitude of the preceding tail-I calculated by exponential fitting of the tail-I of the 1st Ih. **(H)** Normalized inhibition of Ih obtained before (Control) and after optogenetic stimulation (*n* = 7). Paired *t*-test: *p < 0.001. **(I)** Outward shift of baseline-I obtained before (Control) and after optogenetic stimulation (*n* = 7). Paired *t*-test: *p = 0.008. **(J,K)** The repetitive 1 s optogenetic stimulations at short intervals smaller than 10 s induced a cumulative increase in Ih inhibition only transiently and was followed by a forced termination of the Ih inhibition. Ih amplitude was measured for the response evoked approximately 10 s after the respective stimulation. If 10 s after stimulation was just in between the two responses, the average amplitude of the two responses was used as a response for the stimulation. Note that a short interval of the first and second optogenetic stimulations resulted in a much larger Ih inhibition compared to the case with a longer interval > 20 s. **(L,M)** Significant cumulative increases in normalized inhibition of Ih induced following up to 6–7 repetitive 1 s microstimulations with an interval of 9 s (**M**; *n* = 7), after which the forced termination of Ih inhibition began and the maximal inhibition of Ih was significantly decreased after 10 repetitive microstimulation (**M**; *n* = 7). Two-way repeated measures ANOVA: *p* < 0.001, followed by Bonferroni *post hoc* test, *p < 0.03 (vs. control; the largest *p*-value is 0.028) and #*p* < 0.002 (vs. 7th stimulation; the largest *p*-value is 0.0012). Vertical scale bars; **(E,F,J,L)** 200 pA. Horizontal scale bars; **(F)** 500 ms: **(J,L)**, 10 s.

**FIGURE 6:**
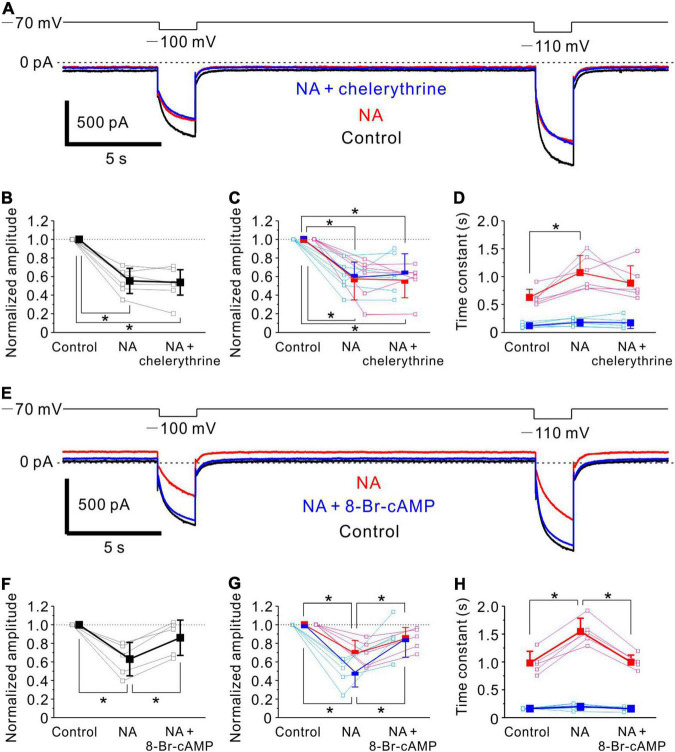
Noradrenaline -induced Ih inhibition in MTN neurons is mediated by cAMP signaling. **(A)** Ih responses evoked by negative voltage pulses stepped from –70 to –100 mV and –110 mV in an MTN neuron obtained under control conditions (black), during application of NA (red) and during application of NA and chelerythrine (blue). Chelerythrine had no effect on NA-mediated Ih inhibition. **(B)** Normalized amplitude of Ih at respective conditions in **(A)** (control, 1.0; NA, 0.55 ± 0.14; NA + chelerythrine, 0.54 ± 0.14; *n* = 6). One-way repeated measures ANOVA: *p* < 0.001, followed by Bonferroni *post hoc* test, *p < 0.001 (control vs. NA), and *p < 0.001 (control vs. NA + chelerythrine). **(C)** Normalized amplitude of fast and slow components (blue and red squares, respectively) at respective conditions in **(A)** (*n* = 6). Normalized amplitude of fast component: control, 1.0; NA, 0.59 ± 0.17; NA + chelerythrine, 0.63 ± 0.22. One-way repeated measures ANOVA: *p* < 0.001, followed by Bonferroni *post hoc* test, *p < 0.001 (control vs. NA) and *p = 0.001 (control vs. NA + chelerythrine). Normalized amplitude of slow component: control, 1.0; NA, 0.57 ± 0.23; NA + chelerythrine, 0.56 ± 0.19. One-way repeated measures ANOVA: *p* < 0.001, followed by Bonferroni *post hoc* test, *p < 0.001 (control vs. NA) and *p < 0.001 (control vs. NA + chelerythrine). **(D)** Time constants of fast and slow components (blue and red squares, respectively) at respective conditions in **(A)** (*n* = 6). Time constant of fast component: control, 0.12 ± 0.04 s; NA, 0.18 ± 0.07 s; NA + chelerythrine, 0.17 ± 0.10 s. One-way repeated measures ANOVA: *p* = 0.194. Time constant of slow component: control, 0.63 ± 0.15 s; NA, 1.07 ± 0.31 s; NA + chelerythrine, 0.88 ± 0.31 s. One-way repeated measures ANOVA: *p* = 0.003, followed by Bonferroni *post hoc* test, *p = 0.004. **(E)** Ih responses evoked by negative voltage pulses stepped from –70 to –100 mV and –110 mV in an MTN neuron obtained before (Control; black), during application of NA (red), and during application of NA and 8-Br-cAMP (blue). 8-Br-cAMP reversed NA-induced inhibition of Ih. **(F)** Normalized amplitude of Ih at respective conditions in **(E)** (control, 1.0; NA, 0.63 ± 0.18; NA+8-Br-cAMP, 0.86 ± 0.19; *n* = 5). One-way repeated measures ANOVA: *p* = 0.002, followed by Bonferroni *post hoc* test, *p = 0.002 (control vs. NA) and *p = 0.023 (NA vs. NA + 8-Br-cAMP). **(G)** Normalized amplitude of fast and slow components (blue and red squares, respectively) at respective conditions in **(E)** (*n* = 5). Normalized amplitude of fast component: control, 1.0; NA, 0.49 ± 0.16; NA + 8-Br-cAMP, 0.85 ± 0.20. One-way repeated measures ANOVA: *p* = 0.002, followed by Bonferroni *post hoc* test, *p < 0.001 (control vs. NA) and *p = 0.041 (NA vs. NA + 8-Br-cAMP). Normalized amplitude of slow component: control, 1.0; NA, 0.69 ± 0.14; NA + 8-Br-cAMP, 0.85 ± 0.12. One-way repeated measures ANOVA: *p* = 0.001, followed by Bonferroni *post hoc* test, *p < 0.001 (control vs. NA) and *p = 0.034 (NA vs. NA + 8-Br-cAMP). **(H)** Time constants of fast and slow components (blue and red squares, respectively) at respective conditions in **(E)** (*n* = 5). Time constant of fast component: control, 0.16 ± 0.01 s; NA, 0.20 ± 0.06 s; NA + 8-Br-cAMP, 0.16 ± 0.04 s. One-way repeated measures ANOVA: *p* = 0.333. Time constant of slow component: control, 0.98 ± 0.21 s; NA, 1.55 ± 0.24 s; NA + 8-Br-cAMP, 1.00 ± 0.13 s. One-way repeated measures ANOVA: *p* < 0.001, followed by Bonferroni *post hoc* test, *p < 0.001 (control vs. NA) and *p < 0.001 (NA vs. NA + 8-Br-cAMP).

### Electrophysiological Recordings and Microstimulation

Using two Axopatch 200B preamplifiers (MDS Analytical Technologies, Sunnyvale, CA, United States), dual whole-cell recordings under voltage-clamp and current-clamp conditions were respectively made from MTN and LC neurons that were viewed under Nomarski optics (BX51WI; Olympus, Tokyo, Japan). The distance between the two tips of patch pipettes for dual recordings from a pair of neurons in MTN and medially adjoining dorsal LC was between 100 and 200 μm. The recording chamber, with a volume of 1.0 ml, was continuously perfused with the extracellular solution (N-aCSF) at a flow rate of 1.0–1.5 ml/min. The internal solution of the patch pipettes had the following ionic composition (in mM): 123 K-gluconate, 18 KCl, 10 NaCl, 2 MgCl_2_, 2 ATP-Na_2_, 0.3 GTP-Na_3_, 10 HEPES, and 0.2 EGTA; with pH 7.3 adjusted with KOH. The membrane potential values given in the text were corrected for the junction potential (10 mV) between the internal solution for the whole-cell recording (negative) and the standard extracellular solution. The pipette resistances were 4–6 MΩ. The series resistance was < 10 MΩ. All recordings were made at RT. Records of currents and voltages were low-pass filtered at 5 kHz (3-pole Bessel filter), digitized at a sampling rate of 10 kHz (Digidata 1322A, MDS Analytical Technologies) and stored on a computer hard disk.

The amplitudes of Ih were measured 10–20 ms before the offset of the negative voltage step (Kang et al., 1994; Tanaka et al., 2003). To isolate Ih or to prevent Ih from being contaminated with other ionic currents, any blocker was not used because of two reasons. First, we did not measure tail current of Ih because low voltage activated currents may contaminate with the tail-Ih at the offset of negative command pulses. Secondly, we measured the maximal amplitude of Ih just before the offset of the negative command pulses because the amplitude of non-Ih currents is less than 6% of the maximal Ih measured just before the offset of Ih (Kawasaki et al., 2018). Furthermore, the small contamination of Ih with inward rectifier and leak K^+^ currents did not cause any problems when the conductance of voltage-dependent HCN was measured because inward rectifier and leak K^+^ currents are not voltage-dependent, and their conductances are constant and very small.

With a tungsten microelectrode with an impedance of 1 MΩ at 5 kHz, microstimulation at an intensity of < 2 V (2.0 μA) with a negative pulse (0.2 ms duration) was applied at 100 Hz for 0.1 s (Figure 4) or 20 Hz for 1 s (Figure 5) in LC. The current spread may be less than 30–40 μm for such microstimulation (Stoney et al., 1968; Jankowska and Roberts, 1972; Kang et al., 1994).

### Viral Vector Transduction of Locus Coeruleus Neurons With ChannelRhodopsin2

To allow optogenetic activation of LC neurons, a Canine-Adenoviral vector (CAV) was used (CAV-PRS-ChR2-mCherry) (Li et al., 2016). The prolyl tRNA synthetase (PRS) promoter in this vector has been previously demonstrated to achieve LC-specific expression by harnessing the Phox2B transcription factor, which drives endogenous dopamine-β-hydroxylase (the final enzyme in the synthesis pathway for NA).

Stereotaxic injection of the CAV vector was made into the dorsal pons in the region of the LC of PD 19 Wistar rats using an established protocol (Li et al., 2016). Rats were anesthetized (intraperitoneally) with ketamine (5 mg/100 g, Vetalar; Pfizer, New York, NY, United States) and medetomidine (30 μg/100 g, Domitor; Pfizer) until loss of paw withdrawal reflex. The animal was placed in a stereotaxic frame and core temperature was maintained at 37°C using a homeothermic blanket (Harvard Apparatus, Holliston, MA, United States). Following a craniotomy (from bregma: –1 mm AP, +1.1 mm ML), a microinjection pipette was advanced into the brain with a 10° rostral tilt. Two hundred nanoliters of vector was injected at 4.6, 4.9. 5.2, and 5.5 mm depth. The titre of vector used was 2.15 × 10^11^ physical particles/ml. Aseptic surgical techniques were used throughout.

### Optogenetic Stimulation

Brain slices including the LC were prepared from PD 27–31 Wistar rats (8–12 days after CAV injection). Rats were terminally anesthetised with isoflurane before decapitation. The brain was dissected, and a tissue block containing the medulla and pons was prepared, in ice-cold dissection aCSF (identical to recording aCSF below, except NaCl was reduced to 86 mM, and sucrose added at 58.4 mM). The tissue block was glued, rostral face down, to the stage of a Dosaka LinearSlicePro vibratome (DSK, Japan), and 300 μm thick coronal slices were cut. Slices were incubated in carbogen bubbled recording aCSF at 37°C for 1 h prior to recordings (in mM): 126 NaCl, 2.5 KCl, 26 NaHCO_3_, 1.25 NaH_2_PO_4_, 2 MgCl_2_, 2 CaCl_2_ and 10 D-glucose, pH 7.3, and osmolality 290 mOsm/l.

For recordings, the slices were placed in the perfusion chamber of an upright fluorescence microscope (DMLFSA, Leica Microsystems, Heidelberg Germany) and continuously perfused with recording aCSF at a ∼2 ml/min flow rate at 30°C. Patch pipettes (4–6 MΩ) were filled with the internal solution as described above. The membrane potential values given in the text were corrected for the junction potential (10 mV). Channelrhodopsin-2 (ChR2) expression neurons were identified using epifluorescence illumination (Chroma filterset 41034) *via* membrane-bound mCherry fluorescence. Recordings were made in voltage clamp mode (Axon Multiclamp 700A, Molecular Devices, San Jose, CA, United States). All recordings were low-pass filtered with a 3 kHz cut-off frequency, and digitized at a sampling rate of 10 kHz (power1401, Cambridge Electronic Design, Cambridge, United Kingdom) and stored on a computer hard disk using Spike2 software (CED). For optogenetic stimulation, light was pulsed onto the cells (5–20 mW, 20 pulses of 4 ms duration at 20 Hz) using a focally placed optical fiber (400 μm tip diameter) proximate to the LC pigtailed to a 473 nm LED source (Doric Lenses, Quebec, QC, Canada) using stimulation scripts implemented in Spike2.

### Drug Application

Atipamezole (a synthetic α_2_-AR antagonist), 8-bromoadenosine 3’,5’-cyclic monophosphate sodium salt (8-Br-cAMP; a membrane-permeable cAMP analog), and chelerythrine chloride [a highly specific protein kinase C (PKC) inhibitor] were bath-applied at 1, 500, and 10 μM, respectively. All chemicals were purchased from MilliporeSigma unless specified otherwise.

### Statistical Analysis

Statistical analysis was performed using STATISTICA10J (StatSoft Japan, Tokyo, Japan). Numerical data were expressed as the mean ± SD. The statistical significance was assessed using *t*-test and/or ANOVA followed by Bonferroni *post hoc* test. Statistical results were given as a specific *p*-value, except when *p* was smaller than 0.001. *p* < 0.05 was considered statistically significant.

## Results

### Noradrenergic Projections From Locus Coeruleus Neurons Onto Mesencephalic Trigeminal Nucleus Neurons and Expression of α_2A_-Adrenergic Receptors on Mesencephalic Trigeminal Nucleus Neurons

First, the noradrenergic projections from LC neurons to MTN neurons were investigated by immunohistochemistry. TH-positive LC neurons project noradrenergic fibers throughout the neuraxis, including MTN which is located dorso-laterally adjacent to the LC nucleus (Paxinos and Watson, 2013). Histological identification of MTN neurons in the mesencephalic region was done by staining with advillin which is a marker for primary sensory neurons (Hasegawa et al., 2007). Advillin-positive MTN neurons were located medially adjoining LC, and occasionally found to be intermingled with the soma of noradrenergic LC neurons (Figure 1A). MTN neurons received noradrenergic projections from LC, as reported previously (Takahashi et al., 2010). Indeed, many TH-positive beaded axonal projections to an MTN neuron can be seen in a z-stack of eight images (Figure 1B) of the area enclosed with a square (Figure 1A) when visualized at 100x magnification. Four terminal-like swellings arising from such TH-positive beaded axons indicated with filled arrow head (Figure 1C) were found to surround the MTN neuron almost without any direct contacts when examined at respective single plane images. The distance between the closest terminal-swelling and MTN soma membrane was larger than 1 μm. In the single plane images either over the second (Figure 1Ca) or under the fifth plane image (Figure 1Cd), there were no terminal-like swellings in close proximity to the MTN neuron. These results were in good agreement with a previous study (Takahashi et al., 2010) which demonstrated that there were almost no synapse-like contacts between terminal-like swelling arising from LC neurons and somata of MTN neurons.

Volume transmission of NA from LC to MTN neurons would take place from the terminal swellings to the cell surface receptors, the distance between which was larger than 1 μm (see Figure 1C). We further examined if there is any difference in the distribution of TH and DBH immunoreactive terminals of LC neurons. We found that immunoreactivities for TH and DBH were almost the same in the somata of LC neurons, while fine terminal arborizations in the MTN region were not immune-positive to DBH but positive to TH (Supplementary Figure 1). This suggests that only small vesicles containing NA produced in the soma are conveyed through short axons to be released from the terminals surrounding MTN neurons. We next examined whether MTN neurons express α_2A_-ARs. In MTN neurons, the intense immunoreactivities for advillin, a sensory neuron-specific protein (Hasegawa et al., 2007), and α_2A_-ARs were seen along the plasma membrane and partly in the cytoplasm (Figure 1D).

### Volume Transmission of Noradrenaline From a Single Locus Coeruleus Neuron to an Mesencephalic Trigeminal Nucleus Neuron

To investigate the activity-dependent nature of volume transmission of NA from LC to MTN neurons, we first performed the dual-whole cell recordings from LC and MTN neurons and examined the effects of spiking of the LC neuron on the h-current (Ih) evoked in the simultaneously recorded MTN neuron. Ih was repeatedly evoked by a 1.5-s negative voltage pulse applied from −70 to −100 mV in the MTN neuron every 30 s, while at 4.5 s before the voltage pulse, a 1-s depolarizing current pulse was applied every 30 s to induce a spike train approximately at 20 Hz in the LC neuron after control Ih was evoked six times (Figure 2A). Because the spike train at 20 Hz is usually followed by a long-lasting (∼30 s) afterhyperpolarization, presumably due to autoinhibition (Arima et al., 1998), together with Ca^2+^ dependent K^+^ channel activity (Osmanovic and Shefner, 1993; Supplementary Figure 2), we usually applied current pulses at an inter-pulse interval of 30 s. These six responses of Ih in the MTN neuron without preceding spikes in the LC neuron showed an almost constant amplitude of Ih. However, as shown in Figures 2A,B (compare black trace with blue and red traces), Ih gradually decreased in amplitude and reached a minimal level after a 20 Hz spike-train was repeatedly induced in the LC neuron.

After the simultaneous whole-cell recording, the LC and MTN neurons were identified with Lucifer yellow, and the LC neuron was found to be TH-positive among other TH-positive neurons (Figure 2C). This TH-positive LC neuron was electrophysiologically characterized as showing a delayed spiking with or without the immediate-onset spike in response to depolarizing current pulses (Figure 2B, upper blue and red traces showing LC firings), which is in good agreement with those of LC neurons reported previously (Masuko et al., 1986; van den Pol et al., 2002; Wagner-Altendorf et al., 2019). Thus, following the repetitive release of NA around the recorded MTN neuron, Ih evoked in the MTN neuron was gradually suppressed by repetitive activation of AR every 30 s and reached a plateau level.

### Nature of Volume Transmission of Noradrenaline From a Locus Coeruleus Neuron With Periodic Phasic Activity

Considering the long inter-stimulus interval (30 s), the apparent saturation of inhibitory effects of LC activity on Ih in MTN neurons is not likely due to the simple saturation of NA around AR. Because the whole time course of the volume transmission accompanied by GPCR activation may be very slow, the repetitive activation of LC neurons may cause cumulative increases in the signal transduction to gradually decrease Ih in MTN neurons. Therefore, we analyzed this cumulative increase quantitatively. As illustrated in Figure 2D, with an increase in the cumulative spike number in the LC neuron, Ih decreased in amplitude consistent with the outward shift of the baseline current, reflecting the steady state Ih at −70 mV. The normalized inhibition of Ih was plotted against either the cumulative spike number or the number of current pulse injection (Figure 2E). These relationships were fitted with various functions and were found to be best described by Hill equation with an nH of 1.49 or 1.44 (correlation coefficient = 0.99). The nH would represent the efficacy of signal transduction in response to LC spike inputs, and therefore can quantitatively characterize the volume transmission.

In a total of 55 paired recordings from LC and MTN neurons, 17 pairs of LC and MTN neurons were found to have positive connections, in which Ih in MTN neurons was significantly inhibited by the activity of LC neurons. Of the 17 positive pairs, effects of repetitive activation of LC neurons were successfully examined in 7 MTN neurons, while effects of antagonist for α_2_-AR and of the microstimulation of LC were successfully examined in 4 and 6 paired recordings, respectively. The maximal normalized inhibition of Ih was 0.19 ± 0.07 and of nH was 1.48 ± 0.32 (*n* = 6) with one exception that showed a smaller nH (0.6), with its maximal inhibition largely diverged from the calculated value (Figure 2F). The averaged correlation coefficient was 0.98 ± 0.01. Thus, our analysis revealed that the volume transmission of NA from LC neurons to MTN neurons, which was induced following repetitive activation of LC neurons every 30 s, can be best described by Hill equation with an nH of approximately 1.5. This value was in good agreement with the averaged value of nH (1.53 ± 0.10) obtained in the relationship between the dose of NA and normalized inhibition of Ih in respective MTN neurons (*n* = 8, Supplementary Figure 3). Because the amplitude of Ih was decreased in a dose-dependent manner with *K*_i_ = 16 μM and a dynamic range (10–90% inhibition) spanning more than 23-fold range of concentration (3–70 μM), it is suggested that the basal level of cyclic monophosphate (cAMP) reflecting tonic activity of adenylate cyclase is high enough for NA to inhibit Ih in MTN neurons in a dose-dependent manner.

These results suggest that the Hill-type relationship obtained in response to repetitive activation of spike trains in LC neurons represents the nature of the volume transmission of NA from LC to MTN neurons and can be a measure of volume transmission (see section “Discussion”).

### α_2_-Adrenergic Receptors Mediated Volume Transmission of Noradrenaline From Locus Coeruleus Neurons to Mesencephalic Trigeminal Nucleus Neurons

In the next experiments, we have studied whether the inhibition of Ih in MTN neurons by the spike activity of LC neurons is abolished by α_2_-AR antagonists. Control responses of Ih were evoked by the negative voltage pulses stepped from –70 to –90 up to –130 mV by 10 mV steps (Figure 3B, black traces of Ih). After control, 5 responses of Ih were obtained in the MTN neuron, and a train of spikes at 20 Hz was induced in the simultaneously recorded LC neuron (Figure 3A, black trace; 1st spike train). Similar spike trains were repetitively induced 20 times every 30 s. After evoking the 20th spike train (Figure 3A, red trace), Ih was examined at respective voltages. As seen in the superimposed traces (Figure 3B, bottom traces), Ih responses were invariably suppressed at any voltage steps, consistent with the previous results (Figure 2). Next, in the presence of atipamezole, Ih responses that were evoked after spike trains were repeatedly evoked 20 times every 30 s were larger than those evoked after 20 spike trains in the absence of atipamezole as shown in the superimposed traces in Figure 3D (bottom traces). Thus, the inhibition of Ih by the activity of LC neurons was abolished after applying atipamezole. Voltage-dependent conductance curve of Ih was significantly shifted in the left direction following LC activation [two-way ANOVA: *F*(2,8) = 77.8, *p* < 0.001, followed by Bonferroni *post hoc* test, *p < 0.001], while such leftward shift was reversed and abolished by atipamezole (Bonferroni *post hoc* test: #*p* < 0.001; Figure 3E). In a total of four paired recordings (of 17 total positive paired recordings) with multiple step pulse responses, a similar leftward shift of conductance-voltage (G-V) relationship was observed following repetitive LC activation [two-way repeated measures ANOVA: *F*(8,24) = 3.73, *p* = 0.006, followed by Bonferroni *post hoc* test, *p < 0.002 (the largest *p* value is 0.0015)], while atipamezole reversed and abolished such a leftward shift of the G-V relationship [Bonferroni *post hoc* test: #*p* < 0.001 (the largest *p*-value is 0.0002)] (Figure 3F). Taken together, these results clearly indicate that the inhibition of Ih in MTN neurons was mediated by the activation of α_2_-AR through volume transmission of NA from single LC neurons.

### Volume Transmission of Noradrenaline From Locus Coeruleus Neurons That Show Phasic-Tonic Activity

A similar nature of volume transmission was also observed following activation of multiple LC neurons with microstimulation. Dual whole-cell recordings were performed from LC and MTN neurons (Figures 4A–D) with two patch pipettes, and a stimulating electrode was placed in LC (Figure 4F). In response to microstimulation applied in LC every 30 s, just 0.5 s before the onset of the voltage pulse to evoke Ih in MTN neurons, the LC neuron displayed a spontaneous firing at approximately 0.8 Hz, while Ih was simultaneously induced in the recorded MTN neuron (Figure 4A, *1). The first microstimulation applied in LC induced a transient increase in spike firing, which did not cause any marked inhibition of Ih (Figure 4B, *2), indicating that the latency to the onset of Ih inhibition is longer than ∼2 s. However, the fourth microstimulation induced a persistent firing at 10–20 Hz, and a clear inhibition of Ih was simultaneously observed in the MTN neuron (Figure 4C, *3). Without microstimulation after the fourth one, the LC neuron kept displaying a tonic firing at approximately 5–10 Hz (Figure 4D, *4), resulting in a further inhibition of Ih in the MTN neuron (Figure 4D, bottom trace). Thus, such tonic firing activities in the LC neuron appeared to cause a progressive inhibition of Ih (Figure 4E). Because microstimulation may have activated other LC neurons and the simultaneously recorded LC neuron around the stimulating electrode, the normalized inhibition of Ih would not necessarily reflect the activity of the simultaneously recorded LC neuron. Therefore, the normalized inhibition of Ih was plotted against the time or the number of episodes (Figure 4G) which yielded a better correlation (*r* = 0.99) than what was plotted against the number of spikes in this LC neuron (*r* = 0.96). Similar results were obtained in 6 paired recordings (of 17 positive paired recordings), while in 4 of 6 paired recordings, the nH ranged between 1 and 1.5 with a mean value of 1.24 ± .19. In addition, the mean normalized inhibition of Ih was 0.17 ± 0.10 (Figure 4H). In two of 6 paired recordings, the relationship between the normalized inhibition of Ih and the episode number displayed a dose-response curve with the nH smaller than unity, in which the calculated normalized inhibition exceeded 1, indicating that the estimation of nH may have probably been underestimated due to the higher background tonic activation of α_2_-AR. Distinct from the cases of single LC activation, microstimulation of LC invariably induced the inhibition of Ih in MTN neurons in all the 6 paired recordings examined. Furthermore, the averaged value of the nH seen during persistent/tonic firings in LC neurons in response to microstimulation of LC (1.24 ± 0.19, *n* = 4) was significantly [unpaired *t*-test: *t*(7) = 2.31, *p* = 0.049] smaller than what was obtained (1.57 ± 0.24, *n* = 5) following periodic burst/phasic activations (1-s pulses applied every 30 s) of single LC neurons at ∼20 Hz when cells showing nH ≤ 1 were excluded in the two groups. Persistent/tonic activation of LC neurons at frequencies of 5∼10 Hz induced by microstimulation (Figures 4C,D) would cause an occlusion in the signal transduction to effectively produce Gα-GTP, resulting in a smaller nH.

The inhibitory effects of microstimulation of LC on Ih evoked in MTN neurons were also abolished by atipamezole (Supplementary Figure 4), indicating that the inhibition of Ih in MTN neurons was induced by the activation of α_2_-ARs through volume transmission of NA from LC neurons.

### Optogenetic Stimulation of Locus Coeruleus Revealed a Slow Time Course of Ih Inhibition in Mesencephalic Trigeminal Nucleus Neurons

In a ChR2-expressed LC neuron (Figures 5A,B), a train of optogenetic stimulation (4 ms duration at 20 Hz for 1 s) evoked a train of transient depolarizing potentials, from which a train of spikes was triggered (Figure 5C). After obtaining the control Ih in an MTN neuron, a train of optogenetic stimulation of a population of LC neurons (Figure 5D) was applied 2–5 s before examining the first Ih. Subsequently, Ih that was evoked in an MTN neuron displayed a slow decrease in amplitude and slowly returned to the original amplitude (Figures 5E,F). This change was accompanied by an outward shift of the baseline current with a similar time course (Figure 5G). In 8 MTN neurons examined, the latency, the time-to-peak, and the time-to-termination of Ih inhibition were 2−5 (3.4 ± 1.1; *n* = 8) s, 30–80 (55 ± 17; *n* = 8) s, and 65–120 (98 ± 20; *n* = 7) s, respectively. All these values were approximated as these were obtained by measuring the amplitudes of Ih that were repetitively evoked at intervals greater than 10 s before and after the optogenetic stimulation.

Following optogenetic LC stimulation, the Ih amplitude significantly decreased from −966 ± 213 to −856 ± 177 pA [paired *t*-test: t(6) = –12.73, *p < .001; *n* = 7], consistent with a significant shift of the baseline current in the outward direction from −100 ± 55 to −79 ± 46 pA [paired *t*-test: *t*(6) = –3.90, *p = 0.008; *n* = 7]. The normalized inhibition of Ih was 0.11 ± 0.02 (*n* = 7; Figure 5H) and the outward shift of the baseline current was 20 ± 14 pA (*n* = 7; Figure 5I). The maximal inhibition of Ih caused by activation of multiple LC neurons with single optogenetic stimulation was not markedly different from those observed in response to the first activation of single LC neurons in dual whole-cell recordings (compare the first response in Figure 2 and the response shown in Figure 5). Axons of such LC neurons that were most strongly activated by optogenetic stimulation due to their location in the top surface of slices might have been largely severed in slice preparations. Hence, optogenetic activation of multiple LC neurons may have induced multiple small NA transients around the target MTN neuron in a less synchronized manner, resulting in a slower time-to-peak compared to those caused by activation of single LC neurons (Figure 2).

Although the recovery from the cumulative inhibition of Ih induced in MTN neurons by the repetitive stimulation of LC neurons was not shown (Figures 2, 4), single optogenetic stimulation of LC neurons only transiently inhibited Ih induced in MTN neurons and the amplitude of Ih was restored almost completely in a few minutes after single optogenetic stimulation (Figure 5). Furthermore, atipamezole reversed the Ih inhibition induced by LC activity (Figure 3 and Supplementary Figure 4). These observations completely eliminate the possibility that Ih inhibition was artificially caused.

### Inter-Burst Intervals in Locus Coeruleus Neurons Determine Whether Ih Inhibition Increases in a Hill-Type Manner or Is Transiently Followed by a Forced Termination

Similar to the repetitive 1-s phasic activation of the LC neuron at 20 Hz every 30 s (Figures 2, 3), repetition of 1-s optogenetic stimulation evoking 20 Hz spike trains (Figure 5C) in LC neurons with intervals ranging between 20 and 30 s decreased the amplitude of Ih in a cumulative manner as reflected in the Hill type relationship (Supplementary Figure 5; mean nH = 1.53, *n* = 3). In contrast, however, when the repetitive 1-s optogenetic stimulation was applied at short intervals less than 10 s, Ih inhibition was only induced transiently and was followed by a forced termination of Ih inhibition (*n* = 3, Figures 5J,K). Because it is known that repetitive optogenetic stimulation may cause a more evident rundown of transmitter releases or evoked responses compared to repetitive electrical stimulation (O’Neill et al., 2017), it was examined with microstimulation whether a forced termination can be observed or not. Indeed, a similar forced termination of Ih inhibition following a transient increase in Ih inhibition was observed in 8 LC neurons following microstimulation of LC for 1 s at 20 Hz repeated with an interval of 6 or 9 s (Figures 5L,M). The normalized inhibition of Ih significantly increased and reached a maximal level following 6–7 repetitive microstimulations, after which the forced termination of Ih inhibition began and the maximal inhibition of Ih was significantly decreased after repeating microstimulation ten times [two-way repeated measures ANOVA: *F*(12,72) = 6.42, *p* = 0.008, followed by Bonferroni *post hoc* test, *p < 0.03 vs. control (the largest *p*-value is 0.028) and #*p* < 0.002 vs. 7th stimulation (the largest *p*-value is 0.0012); *n* = 7, Figure 5M].

Taken together, the Hill-type relationship was largely affected by the activity of LC neurons depending on either periodic burst/phasic firings or persistent/tonic firings, while burst/phasic firing occurring at > 0.1 Hz can forcibly terminate Ih inhibition. Thus, the volume transmission of NA from LC to MTN is differentially and dynamically controlled by the activity of LC neurons.

### Noradrenaline-Mediated Ih Inhibition in Mesencephalic Trigeminal Nucleus Neurons Is Caused by Downregulation of cAMP

We next investigated the signaling pathway responsible for the inhibition of Ih by NA in MTN neurons. Control responses of Ih were evoked by the negative voltage pulses stepped from –70 to –100 and –110 mV (Figure 6A, black trace). Following the application of 100 μM NA for 10 min, the baseline current reflecting the steady state Ih at –70 mV was shifted in the outward direction, and the Ih responses evoked at –100 and –110 mV were markedly suppressed (Figure 6A, red trace). The subsequent application of 10 μM chelerythrine, a PKC inhibitor, in addition to NA for 10 min hardly reversed the NA-induced inhibition of Ih (Figure 6A, blue trace). The pooled data analysis revealed that the application of NA significantly shifted the baseline current in the outward direction from –16 ± 36 to 33 ± 44 pA [one-way repeated measures ANOVA: *F*(2,10) = 14.19, *p* = 0.001, followed by Bonferroni *post hoc* test, *p* = 0.002], but the subsequent application of chelerythrine hardly shifted the baseline current (from 33 ± 44 pA to 27 ± 39 pA; Bonferroni *post hoc* test, *p* = 0.920). The application of NA significantly reduced the normalized amplitude of Ih [one-way repeated measures ANOVA: *F*(2,10) = 46.4, *p* < 0.001, followed by Bonferroni *post hoc* test, *p < 0.001], and the subsequent inhibition of PKC activity had no significant effect on the NA-induced Ih inhibition (Bonferroni *post hoc* test, *p* = 0.605; *n* = 6; Figure 6B). Furthermore, the activation kinetics of Ih were compared among the three conditions. Ih was fitted with a double exponential function, and the fast and slow components were subsequently obtained. The application of NA significantly reduced the normalized amplitudes of both fast and slow components [fast component, one-way repeated measures ANOVA: *F*(2,10) = 21.11, *p* < 0.001, followed by Bonferroni *post hoc* test, *p < 0.001; slow component, one-way repeated measures ANOVA: *F*(2,10) = 23.94, *p* < 0.001, followed by Bonferroni *post hoc* test, *p < 0.001], and the subsequent inhibition of PKC activity had no significant effect on both the normalized amplitudes (fast component, Bonferroni *post hoc* test, *p* = 0.735; slow component, Bonferroni *post hoc* test, *p* = 0.660; *n* = 6; Figure 6C). The time constant of the slow component was significantly increased following application of NA [one-way repeated measures ANOVA: *F*(2,10) = 10.54, *p* = 0.003, followed by Bonferroni *post hoc* test, *p = 0.004], while such an increase in the slow time constant was not abolished by the subsequent inhibition of PKC activity (Bonferroni *post hoc* test, *p* = 0.067; *n* = 6; Figure 6D). The time constant of the fast component was hardly affected by NA and the subsequent inhibition of PKC activity [One-way repeated measures ANOVA: *F*(2,10) = 1.83, *p* = 0.194, *n* = 6; Figure 6D].

We then examined the effects of 8-Br-cAMP on the NA-induced inhibition of Ih. The application of 100 μM NA for 10 min shifted the baseline current in the outward direction and suppressed the Ih responses evoked at –100 and –110 mV (Figure 6E, compare black and red traces). The subsequent addition of 500 μM 8-Br-cAMP shifted the baseline current in the inward direction and enhanced the Ih responses evoked at –100 mV and –110 mV (Figure 6E, compare red and blue traces). The pooled data analysis revealed that application of NA significantly shifted the baseline current in the outward direction from –23 ± 62 to 25 ± 64 pA [One-way repeated measures ANOVA: *F*(2,8) = 9.21, *p* = 0.008, followed by Bonferroni *post hoc* test, *p* = 0.011], and the subsequent application of 8-Br-cAMP significantly shifted the baseline current in the inward direction from 25 ± 64 to –14 ± 73 pA (Bonferroni *post hoc* test, *p* = 0.034). The application of NA also significantly reduced the normalized amplitude of Ih [one-way repeated measures ANOVA: *F*(2,8) = 15.97, *p* = 0.002, followed by Bonferroni *post hoc* test, *p = 0.002], but 8-Br-cAMP application, in addition to NA, significantly reversed the inhibition of Ih by NA (Bonferroni *post hoc* test, *p = 0.023; *n* = 5; Figure 6F). The double exponential analysis revealed the significant decrease in the normalized amplitudes of both fast and slow components following NA application [fast component, one-way repeated measures ANOVA: *F*(2,8) = 16.01, *p* = 0.002, followed by Bonferroni *post hoc* test, *p < 0.001; slow component, one-way repeated measures ANOVA: *F*(2,8) = 16.64, *p* = 0.001, followed by Bonferroni *post hoc* test, *p < 0.001], and the subsequent addition of 8-Br-cAMP significantly reversed the inhibition of both the fast and slow components (Bonferroni *post hoc* test, *p = 0.041 and *p = 0.034, respectively; *n* = 5; Figure 6G). The time constant of the slow component was significantly increased following application of NA [one-way repeated measures ANOVA: *F*(2,8) = 42.32, *p* < 0.001, followed by Bonferroni *post hoc* test, *p < 0.001], while it was significantly decreased after the subsequent activation of cAMP activity (Bonferroni *post hoc* test, *p < 0.001; *n* = 5; Figure 6G). The time constant of the fast component was not significantly changed by NA or the subsequent addition of 8-Br-cAMP [one-way repeated measures ANOVA: *F*(2,8) = 1.27, *p* = 0.333; *n* = 5; Figure 6H]. These results indicate that NA-induced Ih inhibition in MTN neurons is not mediated by PKC, but most likely by downregulation of cAMP through the activation of α_2A_-ARs (He et al., 2014).

## Discussion

In the present study, we have revealed for the first time the activity-dependent volume transmission of NA from LC neurons to MTN neurons by performing the dual whole-cell recordings from LC and MTN neurons (Figures 2, 3). Furthermore, activities of multiple LC neurons were manipulated by applying microstimulation (Figure 4 and Supplementary Figure 4) and optogenetic stimulation (Figure 5) during whole-cell recordings in MTN neurons. First, in response to repetitive phasic activation (20 Hz, 1 s) of LC neurons every 30 s, Ih inhibition gradually increased in a Hill-type manner with an nH of approximately 1.5 (Figure 2). Secondly, in response to repetition of microstimulations of LC that caused persistent/tonic firings at 5–10 Hz after evoking transient phasic firings at 10–20 Hz (Figure 3), Ih inhibition also gradually increased in a Hill-type manner but with a smaller nH compared to the phasic activity (compare Figure 4 with Figure 2). These findings indicate that the sensitivity to input LC spikes or the efficacy of the signal transduction induced by a single spike is much higher for the phasic bursts than for the tonic/persistent firings because the average frequency (0.66 Hz) in over 30 s for the phasic bursts (20 Hz for 1 s) is much lower than the 5–10 Hz tonic firing (lasting for 30 s). However, the dynamic range of input signals (Simon et al., 2014) is larger for tonic activity than for the periodic bursts in LC neurons. In contrast, the 20 Hz activity, when induced every 5–10 s, caused only a transient facilitation of Ih inhibition followed by a forcible termination of Ih inhibition (Figure 5). Thus, the nature of the volume transmission of NA from LC to MTN neurons accompanied by α_2A_-adrenergic GPCR activation can be characterized by these three distinct patterns of Ih inhibition in response to the three different patterns of repetitive activation of LC neurons.

### Volume Transmission of Noradrenaline to Activate α_2_-Adrenergic Receptor That Downregulates Ih in Mesencephalic Trigeminal Nucleus Neurons

The processes of NA diffusion from the terminals of LC neurons and subsequent NA accumulation around the target MTN neurons to activate α_2A_-ARs are very transient, probably with a latency of less than 200 ms for the terminals separated even by 10 μm from the target receptors, given the diffusion constant of ∼0.5 μm^2^/ms. The subsequent receptor binding duration may be also transient (∼200 ms) (Beyene et al., 2017). In response to NA binding to α_2A_-adrenergic GPCR, NA accelerates the GDP dissociation rate to facilitate reaction from NA/α_2A_-AR/[G_α_-GDP/G_βγ_] to NA/α_2A_-AR/[G_α_/G_βγ_], which subsequently shifts the equilibrium between NA/α_2A_-AR/[G_α_/G_βγ_] and NA/α_2A_-AR/[G_α_-GTP/G_βγ_] to produce the free G_α_-GTP (Waelbroeck, 1999; Alhadeff et al., 2018). Thereafter, free G_α_-GTP complex starts to interact with adenylate cyclase to inhibit its activity, and simultaneously undergoes auto-hydrolysis of G_α_-GTP complex to slowly terminate such inhibition (0.02/s) (Ross and Wilkie, 2000). Thus, after the binding of NA to α_2A_-adrenergic GPCR, there are at least three processes that are involved in determining the rate of production of free G_α_/GTP complex, subsequently causing a slow Ih inhibition and a slower termination of Ih inhibition. These processes are reflected in the latency (2–4 s), the time-to-peak (30–70 s), and the time to termination (80–120 s) of Ih inhibition in 13 MTN neurons examined (Figure 5). Ih inhibition would occur immediately after the reduction of cAMP concentration because Ih is active under the equilibrium condition between free and bound cAMP to HCN channels. Such slow Ih inhibition following NA transmission was similar to those observed in autonomic ganglionic neurons in PNS (Koketsu and Yamamoto, 1975; Kuba and Koketsu, 1976; Konishi and Otsuka, 1985).

It is known that heteromeric channels of HCN1/HCN2 have voltage and cAMP dependency similar to HCN2 rather than HCN1 (Chen et al., 2001; Ulens and Tytgat, 2001), while HCN1 and HCN2 are modulated mostly by PKC (Reetz and Strauss, 2013) and cAMP (Wainger et al., 2001; Kusch et al., 2011), respectively. Then, it is suggested that Ih in MTN neurons is mediated by heteromeric HCN channels composed of HCN1 and HCN2 (Notomi and Shigemoto, 2004), as Ih evoked in MTN neurons is largely modulated by cAMP but not by PKC (Figure 6).

### Quantification of Volume Transmission

During repetition of phasic spike trains in LC neurons every 30 s, NA is repetitively released while NA concentration would not be cumulatively increased because of the short latency and duration of NA transient (<0.5 s; see above) as opposed to the long interval of 30 s. Nevertheless, Ih inhibition was cumulatively increased with repetition of spike trains. Then, the concentration of free G_α_-GTP complex should have been cumulatively increased with repetition of spike trains every 30 s, while free G_α_-GTP complex very slowly changes into inactive G_α_-GDP due to the GTPase activity of G_α_ (Ross and Wilkie, 2000), given the absence of regulator of G-protein signaling (RGS). Given that the respective NA transients activate a constant number of α_2A_-ARs in MTN neurons, the same amount of NA/α_2A_-AR/[G_α_-GTP/G_βγ_], from which free G_α_-GTP is released, would be repetitively produced in response to repetitive stimulation.

Provided that the production and degradation of G_α_-GTP complex can be approximated to occur simultaneously with two rate constants of α and β, respectively, the probability [*P*(*t*)] of being in a state of free G_α_-GTP complex is given by the special solution of the following 2nd order linear homogenous differential equation:


$$\frac{d^{2}\,P}{d\,t^{2}}+\left(\alpha +\beta \right)\,\frac{d\,P}{d\,t}+\alpha \,\beta \,P=0$$

$$∴P\,\left(t\right)=\left\{ \begin{array}{cc} \frac{\alpha }{\beta -\alpha }\,\left\{\exp\left(-\alpha \,t\right)-\exp\left(-\beta \,t\right)\right\} & \left(t\geq 0\right)\\ 0 & \left(t<0\right) \end{array}\right.$$

where α > 0 and β > 0 (usually α > β). Then, the concentration of free G_α_-GTP complex is proportional to *P*(*t*). Given the simple temporal summation of G_α_-GTP transient in response to repetitive stimulation, the cumulative increase in G_α_-GTP concentration can be calculated as explained in the supporting information (Supplementary Figure 6). Using this mathematical model, we could confirm that the nH measured from the relationship between the spike/stimulus number and normalized inhibition of Ih is almost the same or slightly larger than that measured from the dose-response relationship (Supplementary Figures 3,6) and can be a useful measure of “volume transmission.”

However, when LC neurons displayed persistent/tonic activities, the value of apparent nH became smaller compared to that seen in the slow periodic phasic/burst activity (compare Figure 2 and Figure 4). It is unclear whether tonic/persistent activity served as a background activity that may decrease an apparent nH or caused some occlusion in production of G_α_-GTP complex. A further study is necessary to clarify this mechanism.

In contrast, when the 20 Hz spike train for 1-s duration was repeated at an interval less than 10 s, Ih inhibition was enhanced only transiently and was followed by a rapid termination of Ih inhibition. In response to such a short interval of repetitive activation of LC neurons, G_α_-GTP complex and G_βγ_ are rapidly produced and accumulated around the GPCR. Then, Ih inhibition would be rapidly terminated because G_βγ_ can indirectly facilitate the hydrolysis of G_α_-GTP complex through activation of G_αi_-selective RGS protein (Huang et al., 2014). In a physiological condition, volume transmission from LC neurons may occur in similar manners following LC activity transitions between tonic and phasic firings. In order to firmly establish the three modes of LC activity-dependent volume transmission to differentially modulate Ih in MTN neurons, a further study to investigate the intracellular mechanisms in MTN neurons is necessary.

### Slow Modulation of Ih by Volume Transmission of Noradrenaline Was Necessary to Facilitate Fast Glutamate Synaptic Transmission in Mesencephalic Trigeminal Nucleus Neurons

It is generally considered that noradrenergic fibers arising from LC exert volume transmission onto pyramidal neurons in the PFC. However, 10∼20% of the varicosities arising from noradrenergic fibers are found to form synaptic junctions on the shaft of dendritic spines of pyramidal cells (Seguela et al., 1990; Aoki et al., 1998), while most of α_2_-ARs are found at sites lacking electronmicroscopically identifiable synapses but are invariably near a terminal (Aoki et al., 1998). Therefore, regardless of whether noradrenergic fibers form synaptic junction, activation of extrajunctional α_2_-AR is always in a manner of volume transmission. It should be especially noted that out of 745 terminal-like swellings arising from LC neurons examined in the MTN regions, only 180 swellings (24%) were found in the vicinity of the somata of MTN neurons despite how none of these swellings formed synapse-like contact (Takahashi et al., 2010).

It has been reported that NA was released by exocytosis of a large-dense core vesicle (LDCV) from the soma and dendrites of LC neurons following their phasic firing (Huang et al., 2007), subsequently causing an autoinhibition of LC neurons through activation of G-protein coupled inward rectifier K^+^ currents (GIRKs) (Arima et al., 1998; Torrecilla et al., 2013). This LDCV-mediated transmission is distinct from the classical fast synaptic transmission. However, there was a clear segregation between the two cell groups of LC and MTN neurons (Figure 1A). In addition, many TH-immunopositive fibers project toward and surround MTN neurons (Figures 1B,C). Therefore, it is likely that the majority of MTN neurons receive axonal volume transmission from LC neurons, but rarely the direct LDCV-mediated transmission from the somata/dendrites of LC neurons.

We have previously demonstrated that 8-Br-cAMP application can suppress the burst firing caused by glutamate puff in MTN neurons through an enhancement of Ih (Kawasaki et al., 2018). The present study provides convincing evidence that LC inputs can manipulate the firing pattern of MTN neurons by modulating Ih through α_2_-AR activation. Thus, in MTN neurons, fast glutamatergic synaptic transmission can only function in the presence of slow volume transmission of NA from LC neurons. However, in order to demonstrate the functional significance and dynamics of this volume transmission from LC to MTN neurons, we have to further perform the experiment in *in vivo* conditions by using GPCR-activation based NE (GRAB_NE) sensor (Feng et al., 2019) and electromyographic (EMG) recording of the jaw closing muscles. GRAB_NE sensor can visualize the spread of the NA and the strength of this effect on a larger scale during jaw-closing movement either in stressful condition or induced by optogenetic stimulation of LC.

## Data Availability Statement

The raw data supporting the conclusions of this article will be made available by the authors, without undue reservation.

## Ethics Statement

The animal study was reviewed and approved by the Institutional Animal Care and Use Committee at Osaka University and Seoul National University and the institutional Animal Welfare and Ethical Review Body at University of Bristol.

## Author Contributions

YK and SO designed the research. HT, JW, WK, HK, and OD performed the research. HT, JW, WK, HK, OD, MS, TT, and DK analyzed the data. HT, YK, and SO wrote the manuscript. All authors contributed to the article and approved the submitted version.

## Conflict of Interest

The authors declare that the research was conducted in the absence of any commercial or financial relationships that could be construed as a potential conflict of interest.

## Publisher’s Note

All claims expressed in this article are solely those of the authors and do not necessarily represent those of their affiliated organizations, or those of the publisher, the editors and the reviewers. Any product that may be evaluated in this article, or claim that may be made by its manufacturer, is not guaranteed or endorsed by the publisher.

## Abbreviations

ACSF: artificial cerebrospinal fluid
α_2_-AR: α_2_-adrenergic receptor
DBH: dopamine beta hydroxylase
GIRK: G protein coupled inward rectifier K^+^ current
GPCR: G-protein coupled receptors
HCN: hyperpolarization-activated cyclic nucleotide-gated
Ih: hyperpolarization-activated, cyclic nucleotide-gated HCN channel current
LC: locus coeruleus
LDCV: large-dense core vesicle
MTN: mesencephalic trigeminal nucleus
NA: noradrenaline
nH: Hill coefficient
PB: phosphate buffer
PBS: phosphate buffered saline
PFC: prefrontal cortex
RT: room temperature
TH: tyrosine hydroxylase.
