# Supplementary material for: The Nature of Noradrenergic Volume Transmission From Locus Coeruleus to Brainstem Mesencephalic Trigeminal Sensory Neurons
Source: Front Cell Neurosci. 2022 Apr 26;16:841239. doi: 10.3389/fncel.2022.841239 (PMC9087804; doi:10.3389/fncel.2022.841239)
Supplement: Supplementary file 1 [file Data_Sheet_1.DOCX]

**Supplementary Material**

**1 Supplementary Equation**

**A mathematical model of Ih inhibition brought about by the cumulative increase in Gα-GTP concentration with repetition of stimulation**

Given the simple temporal summation of G_α_-GTP transient, as defined with the formulation of (1), in response to repetitive stimulation with an inter-stimulus interval of *I*, the G_α_-GTP concentration (*P*) at *t* = *τ* (< *I*) after applying the last *n*-th stimulation can be expressed as follows:

$$\sum_{k=0}^{n-1} P\left( \tau+kI \right)= \sum_{k=0}^{n-1} \frac{\alpha}{\beta- \alpha}[\exp\left\{ -\alpha\left( \tau+kI \right) \right\}-\exp\left\{ -\beta\left( \tau+kI \right) \right\}]$$

$$=\frac{\alpha}{\beta- \alpha}\left( \frac{\exp\left( -\alpha\tau\right)\left\{ 1 -\exp\left( -n\alpha I \right) \right\}}{1 -\exp\left( -\alpha I \right)} -\frac{\exp\left( -\beta\tau\right)\left\{ 1 -\exp\left( -n\beta I \right) \right\}}{1 -\exp\left( -\beta I \right)} \right)$$

$$\equiv P_{\text{accumulated}}(n; \alpha,\beta,\tau, I)$$

Then, the inhibition of Ih (*S*_accumulated_) by the repetitive stimulation can be expressed as follows, given that Ih is inhibited dose-dependently by NA with a Hill coefficient of *h*_1_ (= 1.5) and the maximal inhibition of *θ*_1_ (= 0.34) as demonstrated experimentally:

$$S_{\text{accumulated}}(n;\alpha,\beta,\tau, I)=\frac{\theta_{1}{\{MP_{\text{accumulated}}(\alpha,\beta,n;\tau, I)\}}^{h_{1}}}{K_{1}^{h_{1}}+{\{MP_{\text{accumulated}}(n; \alpha,\beta,\tau, I)\}}^{h_{1}}}$$

$$=\frac{\theta_{1}{\{AP_{\text{accumulated}}(n;\alpha,\beta,\tau, I)\}}^{h_{1}}}{1 + {\{AP_{\text{accumulated}}(n; \alpha,\beta,\tau, I)\}}^{h_{1}}} \text{,}$$

where *M* is the maximum concentration of G_α_-GTP and *A* = *M* / *K*_1_.

In response to repetitive stimulation, %inhibition of Ih measured at 6 sec after respective stimulation was saturated mostly after 20 times repetitive stimulation, yielding the maximal %inhibition of Ih which is *r* (= 1.7) times smaller than $\theta_{1}$. Then, *A* can be given as follows:

$A=\left( r-1 \right)^{-\frac{1}{h_{1}}}/P_{\text{accumulated}}(20; \alpha,\beta,\tau, I)$.

In order to perform simulation, parameters *α* and *β* were randomly drawn from the uniform distribution between 0 and 2 sec^-1^, and *I* = 30 sec and *τ* = 6 sec were used to be consistent with the experiment (Fig. 2). Using these values, *S*_accumulated_ (*n*; *α*, *β*, *τ*, *I*) was fitted by the following Hill equation with the least square method.

$$S_{\mathrm{Hill}}(n)= \frac{\theta_{2}n^{h_{2}}}{K_{2}^{h_{2}}+ n^{h_{2}}}$$

We collected 101 parameter sets in which $K_{2}$ ranged between 3.5 and 4.5 to be consistent with the experimental data (Supplementary Figure 6A).

The simulation revealed that there was an inverse relationship between *α* and *β* (Supplementary Fig. 6B) and the Hill coefficient measured in the responses to repetitive stimulations may be slightly larger than that (1.5) obtained by bath application of NA (Supplementary Fig. 6C). Thus, the volume transmission can well be quantified by measuring the Hill coefficient in the responses to repetitive stimulation.

**2 Supplementary Figures**

**
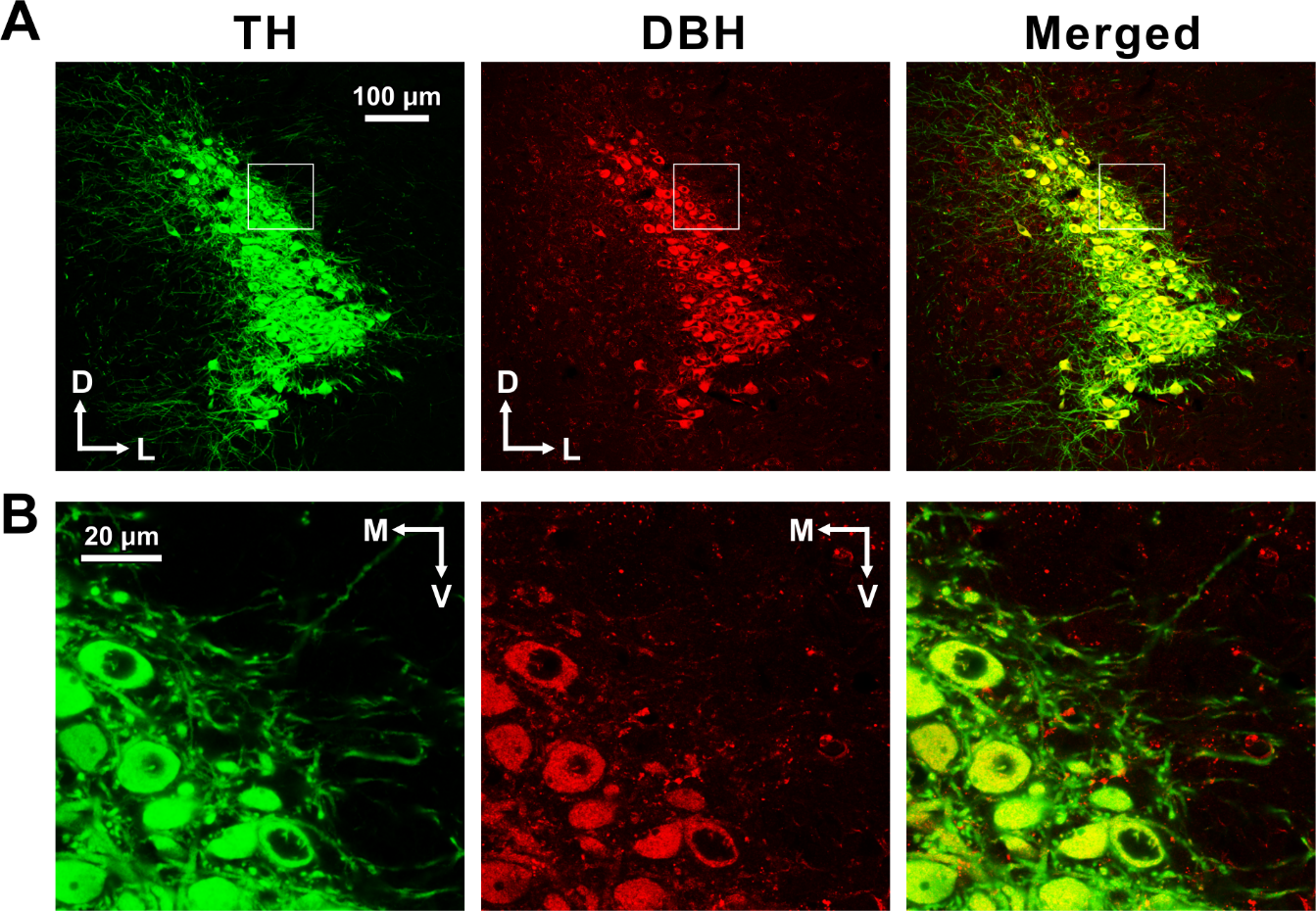
**

**Supplementary Figure 1. Immunoreactivities to TH and DBH in LC.**

(**A**) Confocal images showing the immunoreactivities to TH and DBH, together with a merged one. (**B**) Enlarged images of rectangles shown in **A**. In the MTN region located dorso-laterally adjacent to TH-positive LC, fine terminal arborizations were hardly immuno-positive to DBH but clearly positive to TH.


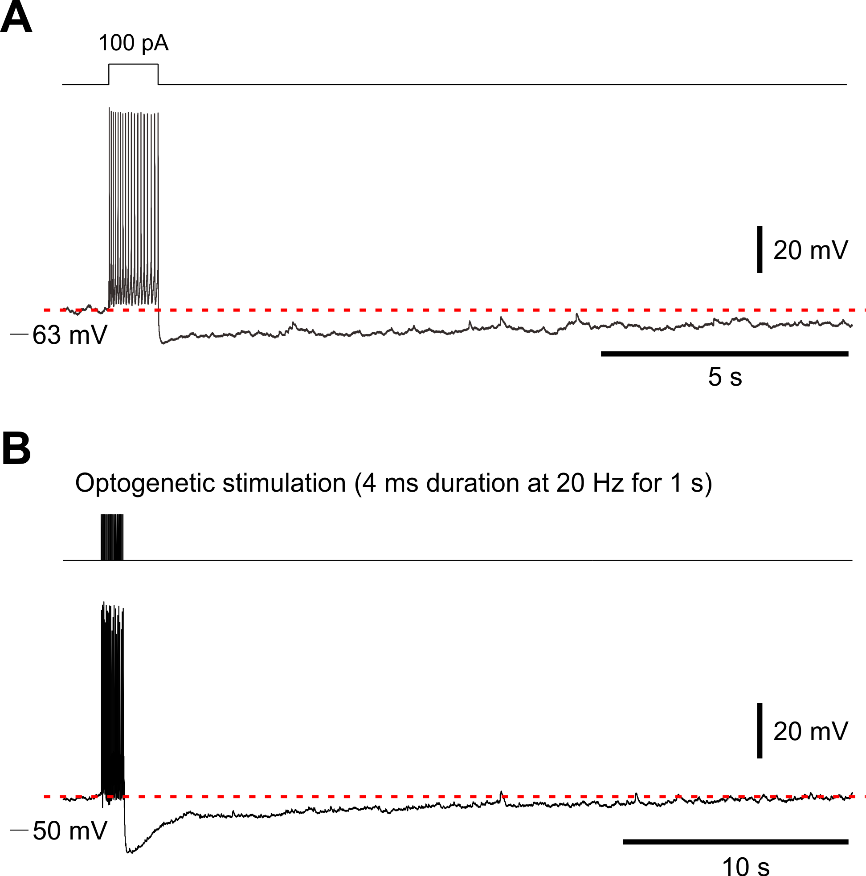


**Supplementary Figure 2. Time course of AHP following a spike train in LC neurons.**

(**A**) A long time course (> 15 sec) of AHP following a 20 Hz spike train (for 1 sec) evoked by a current pulse. (**B**) A long time course (~30 sec) of AHP following a 20 Hz spike train (for 1 sec) evoked by a train of optogenetic stimulation.

**
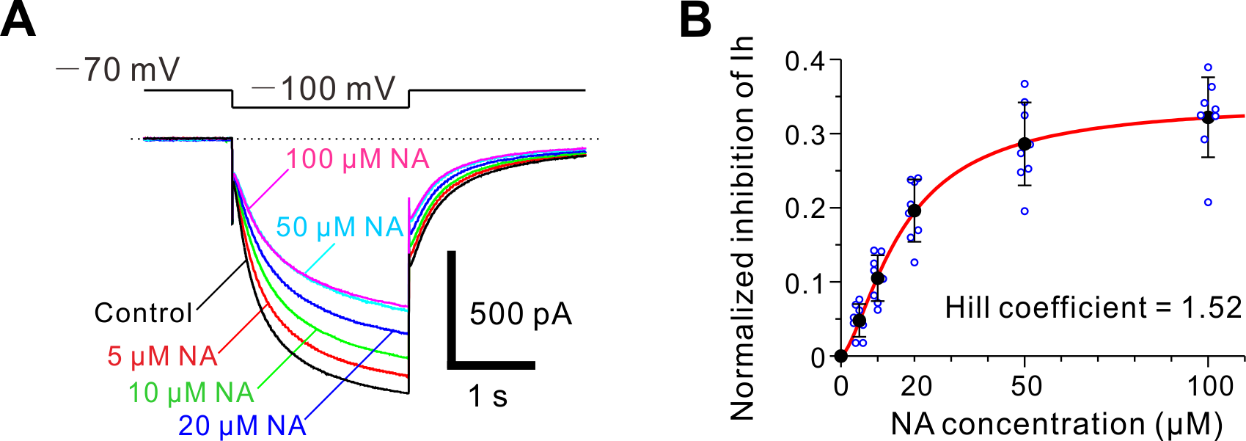
**

**Supplementary Figure 3. The relationship between the dose of NA and %inhibition of Ih in respective MTN neurons.**

(**A**) Superimposed traces of Ih responses evoked by a negative voltage pulse stepped from –70 mV to –100 mV in an MTN neuron obtained before (control) and after application of 5, 10, 20, 50 and 100 μM NA. (**B**) Plotting of normalized inhibition of Ih against NA concentration (n = 8). The red curve was obtained by the Hill equation fitting to the data points of the respective mean values.


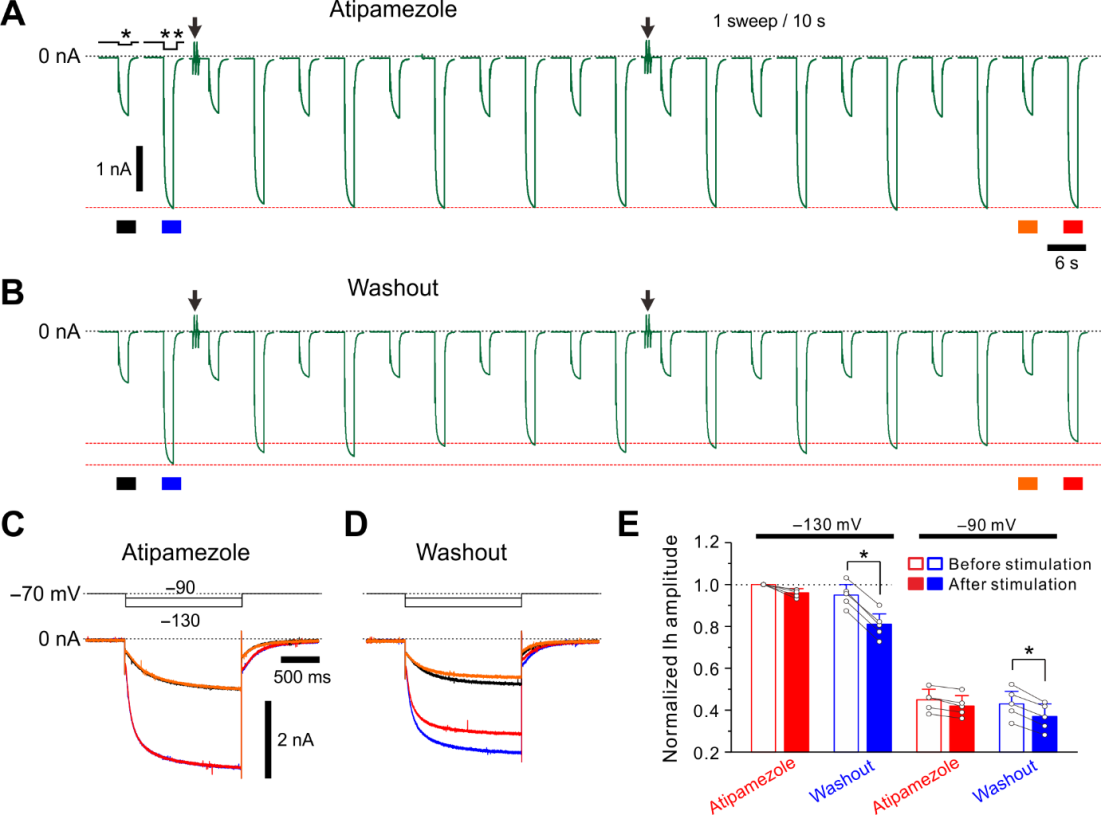


**Supplementary Figure 4. Effects of atipamezole on inhibition of Ih evoked in an MTN neuron by spike activity of multiple LC neurons.**

(**A**, **B**) Ih responses evoked by negative voltage pulses stepped from –70 mV to –90 mV (*) and –130 mV (**) in an MTN neuron obtained in the presence of atipamezole (A) and those obtained after washout of atipamezole (**B**). Arrows indicate microstimulation of LC, which were applied every 30 sec. (**C**, **D**) Enlarged current responses evoked in the LC neuron during the respective time periods indicated with black, blue, orange and red solid bars in **A** and **B**. (**E**) Ih amplitudes normalized to the control Ih obtained before microstimulation in the presence of atipamezole at respective conditions (n = 5). Two-way repeated measures ANOVA; **p* < 0.05.

**
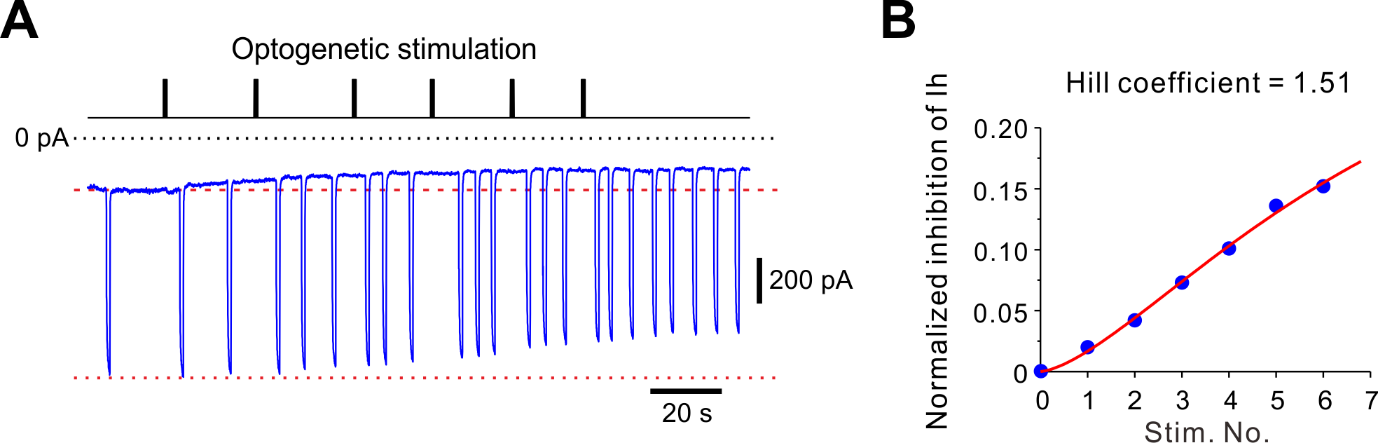
**

**Supplementary Figure 5. Effects of optogenetic stimulation of LC neurons every 20-30 sec on Ih in an MTN neuron.**

**(A**) Repetition of 1-sec optogenetic stimulation evoking 20 Hz spike trains in LC neurons with intervals ranging between 20–30 sec decreased the Ih amplitude in a cumulative manner. (**B**) Plotting of normalized inhibition of Ih against cumulative number of stimulation. The red curve was obtained by the Hill equation fitting to the data points.


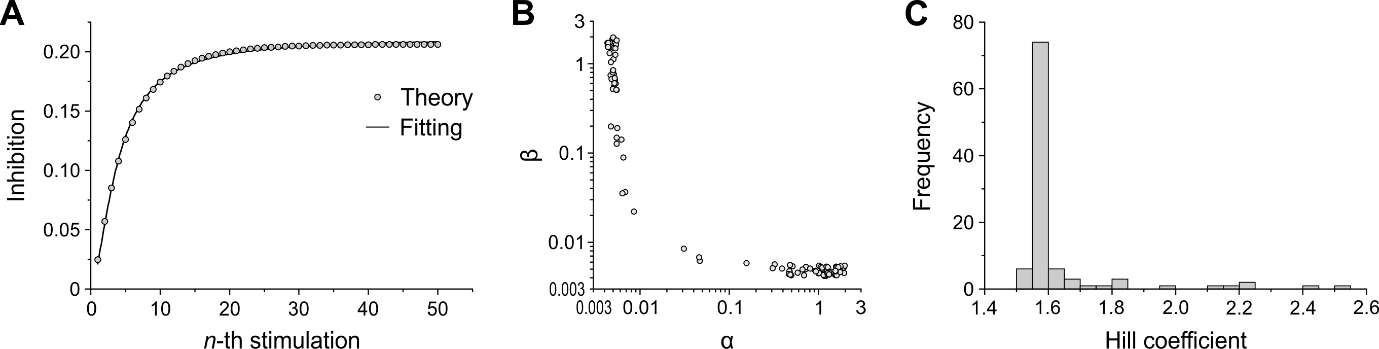
**Supplementary Figure 6. Validation of Hill coefficient measured in the responses to repetitive stimulations by a mathematical model.**

(**A**) The Hill coefficient obtained by Hill fitting is the median of their distribution shown in C. (**B**) An inverse distribution of α and β which satisfy the conditions that *K*_2_ ranges between 3.5 and 4.5 and *θ* = *θ*_1_/*r*. (**C**) Frequency histogram of Hill coefficient in a total of 101 data sets. When α and β took very small values and/or when α < β unlike the values of α and β under the physiological condition, Hill coefficient tended to have a larger value unlike the experimental results.
